# Leveraging Non-Targeted Metabolite Profiling via Statistical Genomics

**DOI:** 10.1371/journal.pone.0057667

**Published:** 2013-02-28

**Authors:** Miaoqing Shen, Corey D. Broeckling, Elly Yiyi Chu, Gregory Ziegler, Ivan R. Baxter, Jessica E. Prenni, Owen A. Hoekenga

**Affiliations:** 1 Boyce Thompson Institute for Plant Research, Ithaca, New York, United States of America; 2 United States Department of Agriculture, Agricultural Research Service, RW Holley Center for Agriculture and Health, Ithaca, New York, United States of America; 3 Colorado State University, Proteomics and Metabolomics Facility, Fort Collins, Colorado, United States of America; 4 United States Department of Agriculture, Agricultural Research Service, Plant Genetics Research Unit, St. Louis, Missouri, United States of America; 5 Donald Danforth Plant Science Center, St. Louis, Missouri, United States of America; Centro de Investigación y de Estudios Avanzados del IPN, Mexico

## Abstract

One of the challenges of systems biology is to integrate multiple sources of data in order to build a cohesive view of the system of study. Here we describe the mass spectrometry based profiling of maize kernels, a model system for genomic studies and a cornerstone of the agroeconomy. Using a network analysis, we can include 97.5% of the 8,710 features detected from 210 varieties into a single framework. More conservatively, 47.1% of compounds detected can be organized into a network with 48 distinct modules. Eigenvalues were calculated for each module and then used as inputs for genome-wide association studies. Nineteen modules returned significant results, illustrating the genetic control of biochemical networks within the maize kernel. Our approach leverages the correlations between the genome and metabolome to mutually enhance their annotation and thus enable biological interpretation. This method is applicable to any organism with sufficient bioinformatic resources.

## Introduction

Assembling increasingly large datasets due to the enhanced efficiency of various phenotyping technologies (e.g. metabolomic and proteomic profiling or nucleic acid sequencing) permits increasingly comprehensive views of biological processes. However, the problem of analysis and visualization in systems biology has led some commenters to question how best to “drink from a fire hose” [1]. Statistical methodologies that are highly inclusive can help solve the dual problem of analysis and visualization [2], [3]. Here, we describe the use of weighted correlation network analysis (WGCNA) as a method to integrate mass spectrometry-based simultaneous of the maize kernel, isolated from a diverse panel of inbred maize varieties previously utilized for genome wide association studies of multiple traits [4], [5], [6], [7], [8]. We assert that the use of such a study panel allows us to leverage the genetic and genomic resources already available to enhance our annotation and analysis of mass spectrometry results. Likewise, this approach also improves the annotation of a genome by providing specific metabolites and chemistries to describe the roles of predicted proteins. This approach relies heavily on software written in the R programming language, which should enable wide adoption by the scientific community due to the lack of associated cost [9].

Our choice of study system was deliberate. Maize has incredible genetic and phenotypic diversity, providing an ideal resource for systems biology studies [10]. Variation in plant yield, composition, and morphological traits has been reported in multiple collections of diverse inbred varieties and related biparental mapping populations [4], [5], [6], [7], [8], [11]. Much has also been learned about the structural and genetic variation within the maize genome [12], [13]. The quality of maize grain is a key factor for breeders and other stakeholders, but the development of biomarkers to assist breeding and transgenic crop improvement remains challenging [14], [15], [16], [17]. Metabolomics, relying on the use of mass signals as markers, provides a rapid approach to characterize related varieties and enable the description of existing and novel quality traits [18], [19]. The aim of metabolomics is to provide a comprehensive and quantitative analysis of a vast number of components in a specific biological sample, and identify as many metabolites as possible [20], [21], [22].

Metabolomic analyses of plants can be especially challenging, as plants contain great chemical diversity especially in secondary metabolites [18], [23]. These secondary metabolites help keep plants’ systems working properly, play roles in the response to genetic or environmental changes, and have powerful physiological effects in humans or animals [20]. Although mass spectrometry-based metabolomics enables the measurement of hundreds or thousands of compounds from a single complex sample, the plant metabolome is still poorly defined and the identification process for specific compounds remains challenging [24], [25], [26], [27], [28], [29]. However, metabolite profiling is not mutually exclusive of statistical genetic and genomics-based approaches [30]; the combination of systems biology strategies is mutually supportive and beneficial.

Genome-wide association studies (GWAS) consider nucleotide variation patterns, relative to population structure, to identify correlations between particular genomic regions and phenotypes. Often, susceptibility to particular diseases or metabolic syndromes is analyzed using GWAS, as this statistical genomics approach is useful in both humans and model systems [7], [31], [32]. GWAS have been applied to metabolomic datasets, to identify SNPs that may be causally linked to particular biochemical processes or pathways [14], [33], [34], [35], [36]. However, in each of these cases the unit of analysis has been individual metabolites, which does little to improve the efficiency of calculation or maximize the benefit of measuring hundreds or thousands of analytes.

Here, we describe non-targeted metabolite profiling of whole maize kernels. The study of grain quality was approached from the perspective of maize as a foodstuff, thus methanolic extracts were isolated from cooked kernels. Our choice of study panel gave us access to more than 1 million SNPs to support GWAS [12]. We applied WGCNA to organize our data into modules that contained multiple markers that also enabled the identification of networks under genetic regulation. This condensation step allowed us to reduce the complexity of the dataset, addressing the multiple testing problem that is endemic to systems biology, and increase computational efficiency. GWAS were applied to weighted averages for each module (hereafter, module eigenvalues), to identify SNPs associated with collections of biochemical markers. We suggest that the WGCNA procedure does not excessively smooth the data, as SNPs correlated with module eigenvalues were significantly correlated with specific compounds assigned to those modules. Finally, module eigenvalues were used in linear regression models to analyze traits that were recalcitrant to GWAS.

## Materials and Methods

### Materials

HPLC-grade acetonitrile, methanol, and formic acid were purchased from Fisher (Pittsburgh, PA); UPLC HSS C18 column (1.8 µm, 2.1 mm×100 mm), sample vials, UPLC column test mix and leucine enkephalin were purchased from Waters (Milford, MA); and all other reagents were available through Sigma (St. Louis, MO), or as indicated.

### Sample Preparation

A maize inbred diversity panel was grown in 2010 on the Musgrave Research Farm of Cornell University (Poplar Ridge, NY)(Flint-Garcia et al., 2005). Duplicated trials were grown using a randomized field design with regular check rows of the B73 accession; 210 of the 282 accessions produced sufficient grain for subsequent analysis, largely due to flowering time issues. Whole maize kernels (n = 50) were covered with an equal volume of 18 megaohm water and autoclaved for 30 minutes to fully cook the grain. Samples were then freeze-dried and ground to a fine flour using a consumer-grade grain mill (KoMo Medium Mill, Pleasant Hill Grain, Hampton, NE). Ground, cooked samples were frozen at −20°C until extracted with a 1∶1 mixture of water and methanol. After 10 min sonication, extracts were centrifuged for 10 min at 4000 rpm. The supernatant was filtered through 0.45 µm filter. Two independent biological replicates were obtained and analyzed, although only one is discussed here.

### UPLC and Mass Spectrometer

Sample injections were performed with an ACQUITY UPLC system (Waters), equipped with a Waters Acquity UPLC HSS C18 column. The samples were injected by means of a 7.5 µL partial loop injection with 3 technical replicates by randomizing all injections. Mobile phase A consisted of 0.1% formic acid in water and mobile phase B contained 0.1% formic acid in acetonitrile. The following gradient was used: 4.5 min 2.4% B, 0.5 min 40% B, 3.5 min 64% B, and 3.5 min 97.6% B. Flow rate was 0.4 ml/min and column temperature was maintained at 40°C. The eluent from the column was delivered to a Xevo G2 TOF (Waters). The mass spectrometer operated in a positive mode using a samples cone voltage of 20 V and a capillary voltage of 2.5 kV with the temperature of source and desolvation at 120°C and 350°C and the flow rate of nitrogen desolvation gas at 850 L/h. Data were acquired in a centroid mode from 50 to1,200 m/z with scan time of 0.2 sec. MS data were collected at a collision energy of 6 V with alternative collection of MSE mode using a ramped collision energy of 20–40 V. Leucine enkephalin was used as the lock mass compound (m/z 556.2771 in positive) and infused at 10 µl/min with a concentration of 1 ng/µl. The lock mass was acquired in all injections of samples to ensure accuracy and reproducibility. The instrument was calibrated using sodium formate at a concentration of 5 mM with mass accuracy within 1 ppm.

### Data Transfer and Statistics Approaches

A variety of statistical procedures were employed to analyze data using R (version 2.13.1) or JMP (version 9, SAS Institute, Cary NC). MarkerLynx (v4.1, Waters) was used to integrate and align MS data points and to convert them into exact mass and retention time signals. Principal component analysis (PCA) was performed using Pareto-scaled data on all detected features for initial charactering the separation of maize variables and checking repeatability for technical replicates. The MarkerLynx generated feature list was somewhat larger than that obtained using XCMS, but WGCNA produced highly similar outcomes from both datasets. To use XCMS to identify and annotate features, raw data files were converted to NetCDF format using the Waters DataBridge software [37]. Peak detection and alignment was performed on both the low and high collision energy channels (MS and MS^E^) using XCMS software (version 1.22.1 [38]). Reconstruction of indiscriminant MS/MS spectra (idMS/MS) was performed as described [37], with exception that rather than utilizing CAMERA groupings, the grouping was centered around the retention time of the feature of interest, with a 2 second window on either side. Reconstructed spectra presented in this paper are supplied as File S1, an msp format spectral file suitable for viewing using NIST MS search program. A correlational filter was then used to find features that demonstrated similar quantitative patterns to the feature of interest. Reconstructed MS and idMS/MS spectra were exported as an ‘.msp’ formatted spectral library using a custom R script. The library was batch searched against the MassBank database [39], and manually searched against the NIST (http://www.nist.gov/srd/nist1a.cfm) and Metlin databases [40]. Identification confidence scores were assigned as described [37]. For multiply charged peptides, the spectra were manually converted to.mgf format, and the precursor ion was manually interpreted based on the MS spectrum. idMS/MS spectra were searched against the NCBInr protein database using a taxonomy filter for maize (version 07/12/12) (43,920 sequence entries) using the Mascot database search engine (version 2.3). Search parameters were set as follows: monoisotopic mass, parent ion mass tolerance of 0.05 Da, fragment ion mass tolerance of 0.1 Da, no enzyme specificity, and variable modification of oxidation of Met.

The XCMS also fills empty cells based on retention time and mass specific signal, thus reducing the frequency of zero values in the dataset. The XCMS generated data matrix, with an intensity value for each feature and each sample, was used as input for the clustering analysis by WGCNA. Weighted correlation network analysis (WGCNA) was produced with the R package, creating unsigned networks where both positive and negative correlations could be clustered into a single module [2], [3]. WGCNA used autoscaled data in order to reduce the dominance of dynamic, high-concentration metabolites (Table S1). Module eigenvalues for each module were calculated for each of the 210 maize accessions, providing a condensed dataset of derived variables for subsequent genetic analysis. Cytoscape, the open source bioinformatics software, was used to illustrate metabolite networks [41].

### Genome-wide Association Study (GWAS)

GAPIT, the genome association and prediction integrated tool (http://www.maizegenetics.net/gapit) was used to perform GWAS and genome prediction. Module eigenvalues from WGCNA were used as maize phenotypic traits in GWAS. The genotypic data are publicly available from panzea.org (http://www.panzea.org/lit/data_sets.html#genos). Previously, the maize diversity panel had been characterized using next-generation sequence analysis such that more than 1 million SNPs across the maize genome are available to characterize the genetic diversity [12]. A kinship matrix was calculated [42] and population structure was modeled as a fixed effect [43]. Significant SNPs (FDR corrected p<0.001 and p<0.05) were identified for each module eigenvalue with a requirement that the SNP be present with ≥5% allele frequency.

## Results

### Non-targeted Metabolite Profiling

**Figure 1:**
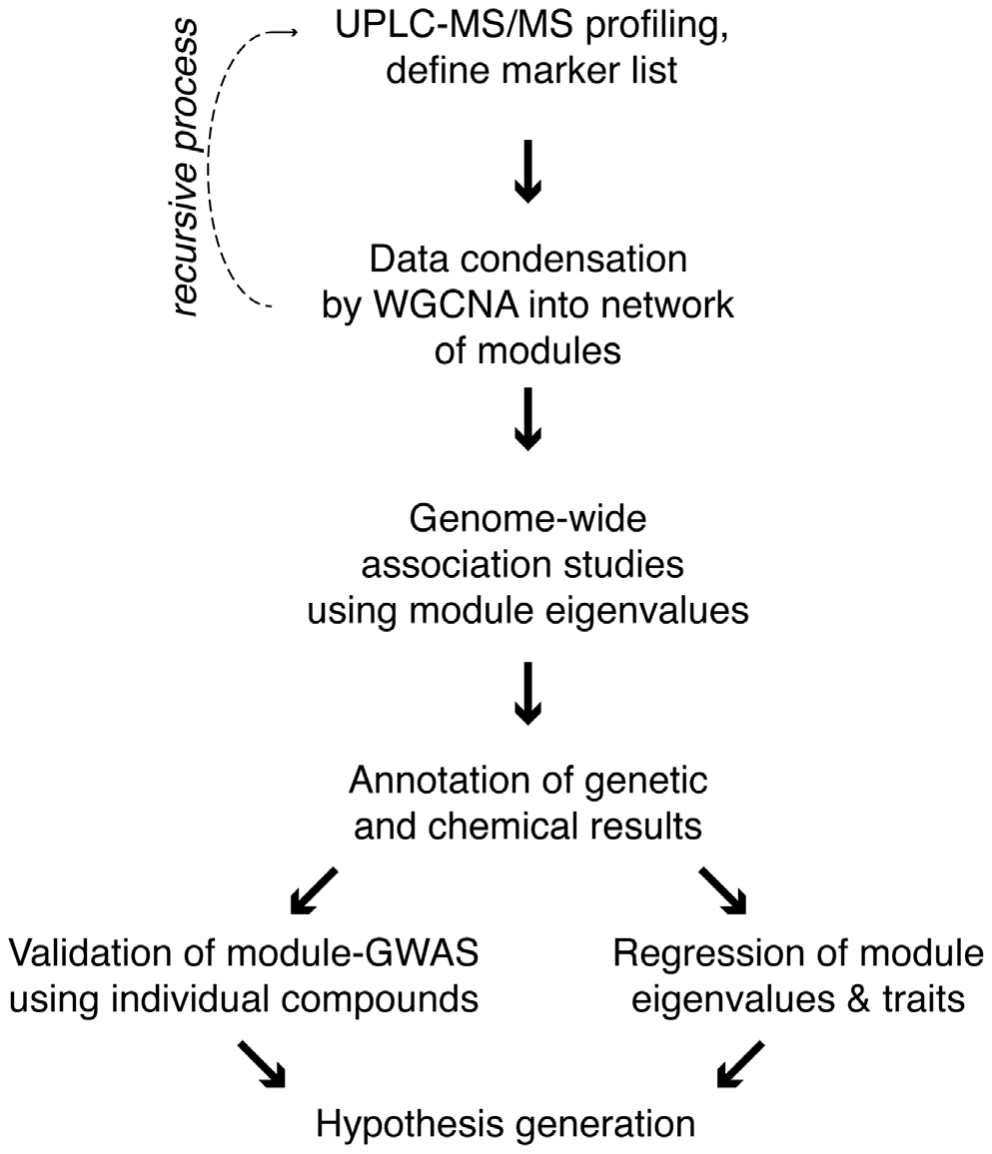
Genomics-assisted chemistry & chemistry-assisted genomics. This flow chart describes the process by which statistical genetics and genomics can enable metabolite profiling to have greater power and impact.

Our long-term goal is to characterize phenotypic variation in maize grain quality and to identify the genetic and environmental factors that influence the metabolomic composition of this important staple food and model plant. This effort will provide information to better describe the existing food supply and also project what new grain quality traits may be achievable in the future using conventional plant breeding. Towards this goal, we chose to use mass spectrometry based non-targeted metabolite profiling of maize meal prepared from cooked, whole kernels (Figure 1). While it may be counterintuitive to treat samples in this way, our dataset represents a genetically diverse sample of a foodstuff that could be consumed by either humans or animals. This choice helps to define the range of normal and acceptable variation within a highly diverse crop plant [17]. More than 8,710 metabolomic features were detected from the whole kernel methanolic extracts (Table S1). Principal component analysis (PCA) gave an initial characterization of the profiling results. PCA explains about 22% of the variance with 2 PC’s (Figure S1). The performance of PCA for this dataset is typical as the composition varies very widely across genetically distant accessions.

### Data Condensation by Weighted Correlation Network Analysis (WGCNA)

One of the endemic problems of systems biology is the multiple testing problem, wherein the number of variables measured dwarfs observations. One potential solution to this problem is to condense the dataset into a smaller number of distinct groups (hereafter, modules), normalizing the issue of observations and variables. WGCNA is an approach to display model network relationships, identifying co-regulated groups of features (hereafter, nodes) such as patterns of gene expression [2]. It can also be used to visualize metabolite networks and increase the comprehensiveness of non-targeted metabolomics [3]. WGCNA describes the relationships between all of the input variables, summarizing the correlation and connectivity of all nodes. The network can be more or less elaborate, depending on the rules set for inclusion into the network. A principal component is calculated for each module for each variety, summarizing the contribution of all nodes included into a particular module, which is referred to by a randomly assigned color. This principal component (hereafter, module eigenvalue) can be used for correlation tests or ANOVA.

**Figure 2:**
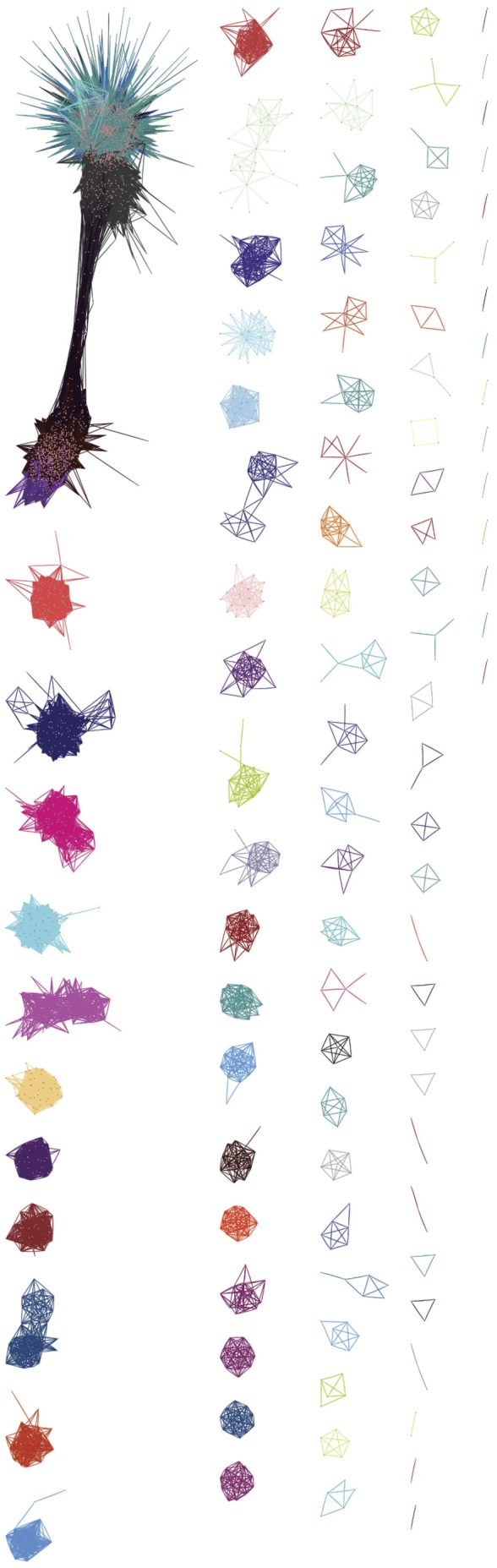
Visualization of maize grain metabolome. This node and edge projection describes the grain metabolome observed in the methanolic extract from 210 inbred line varieties of maize. This network requires a minimum degree of connectivity between any two nodes (i.e. biochemical markers detected by mass spectrometry) that exceeds four standard deviations above the mean connectivity observed between detected markers. According to this threshold, 4,102 nodes are organized into 48 modules each represented by particular color. However, some modules have separated into multiple, distinct clusters as internal connectivity may fall beneath the 4 standard deviation cutoff, such that there are 101 objects in this projection.

For our dataset, 97.5% of the detected molecular features (nodes) could be included in a network with 56 defined modules (Table S2). The network was then pruned to require that the minimum connectivity between nodes exceed 4 standard deviations (SD) above the mean connectivity observed between all nodes. At this threshold, the network contained 48 modules and 4,102 nodes (47% of nodes, 3.1% of the theoretical connections; Figure 2). The network was redefined under even stricter terms, using a 6SD threshold (Table S2). As the modules were defined by the strength of the correlations among members, modules varied in size and membership according to the inclusion threshold. For example, the turquoise module in the initial description had 2,105 nodes and ∼3.73 million edges (Table S2). At the 4SD threshold, the turquoise module reduced to 1,597 nodes with more than 0.62 million edges, while at the 6SD shrinking further to 635 nodes with 40,217 connections. Nodes within the turquoise module were also connected with members of the black module, which likewise contained connections to both the turquoise and purple modules. Other modules were much less elaborate; orange contained 81 nodes in its initial description, 63 nodes at 1 SD, dropping to 9 nodes and 56 connections at 4SD, and disappearing completely at 6SD (Table S2). At the 4SD threshold some modules broke into distinct clusters as connections that helped to define the original module, using the original definitions, dropped below the significance threshold (Figure 2). This facet of the WGCNA procedure represents both a strength and a weakness for the approach. Information can be applied to poorly connected members of a particular module using guilt by association on tightly connected central elements. However, as the module eigenvalues are estimated when the network was initially described, the poorly connected nodes may transmit an excessive degree of variance to these values and perhaps confound downstream applications.

### Module Eigenvalues Drive Genome Wide Association Studies

**Figure 3:**
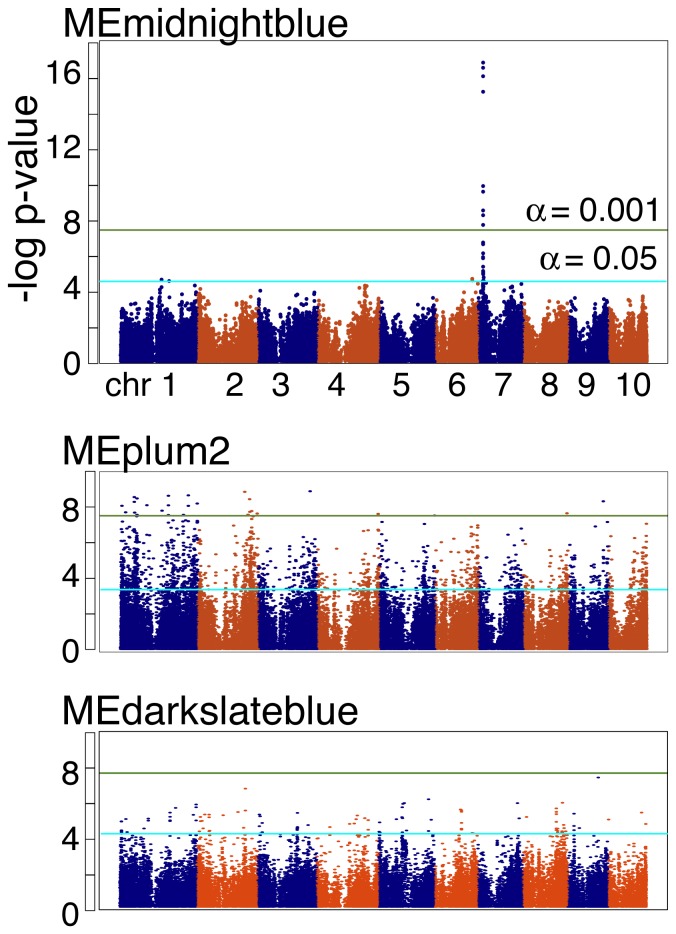
Genome-wide association studies on three module eigenvalues (ME). Nineteen modules returned significantly correlated SNP markers according to GAPIT. Three are shown here. Significance thresholds were empirically calculated for each trait using GAPIT; FDR-corrected p-values at both a conservative (p<0.001; green line) and generous (p<0.05; aqua line) are displayed. MEmidnightblue identified one region of chromosome 7 with high confidence, with a second region of chromosome 1 with lower confidence. MEplum2 identified multiple genomic intervals with high confidence. MEdarkslateblue identified no significant regions at the conservative threshold, but several regions at the lower threshold.

We expected that using WGCNA for the analysis of mass spectrometry based non-targeted metabolite profiling data would accomplish two goals: (1) define the co-regulated networks of metabolites and peptides that contribute to maize kernel quality and composition and (2) reduce the number of variables for downstream analyses. One such analysis is a genome wide association study (GWAS), to correlate particular genomic regions with phenotypes of interest. This approach has already been applied to maize but not on derived variables such as module eigenvalues, so far as we are aware. And while computational resources are improving, conducting GWAS with a SNP dataset as large as that available for the Buckler Diversity Panel using optimized procedures is still a time intensive procedure (0.5 hr/trait or >150 d for the original data) [12], [44]. Module eigenvalues for all 56 modules were analyzed, 19 of which found significant associations (FDR corrected p-value <0.05; Table S2). Modules that were detected under the most stringent membership conditions (>6SD) were more likely to produce significant GWAS outcomes than those present only under lesser requirements (14 of 27 versus 5 of 21; Table S2). However, modules with fewer connections at 4SD were more likely to identify significantly correlated SNPs with GWAS (χ^2^ = 4.56, p = 0.0328). While 4,830 SNPs were identified by GWAS, nearly two-thirds were associated with only two modules (plum2 and salmon). A variety of patterns were observed in the results, ranging from few to many SNPs and wide to narrow distribution across the genome (Figure 3).

The strongest associations between SNPs and module eigenvalues were found with the midnightblue module. Nearly all of the significant SNPs were identified at both conservative and relaxed FDR corrected p-value thresholds and were located in a single region of chromosome 7. Most SNPs were identified with variants of the α-zein 19C2 seed storage protein, a result that is supported by analysis of the mass spectrometry data, which are consistent with a C-terminal peptide derived from α-zein 19C2 protein (Figure S2; Table 1; File S1). The plum2 module gave results as one might optimistically hope for, with a variety of genomic regions identified under conservative significance thresholds. SNPs associated with plum2 included those within a putative mitogen-activated protein kinase, which suggested that signal transduction pathway components were detected by GWAS, and also a cytochrome P450 with significant similarity to flavonoid 3-monooxygenases (Table 1). The darkslateblue module returned far fewer SNPs above the α = 0.001 significance threshold, but many above the 0.05 level. Much like plum2, these SNPs identified a mixture of genes with potential functions while others had no obvious connection to the regulation of maize kernel composition.

**Table 1:** Sample results from genome-wide association studies.

SNP	−log (p-value)	Module/Marker	Gene Annotation
S7_18857356	17.45	Midnightblue*	α-zein precursor 19C2^CDS^
S7_18857356	8.74	1056.528_226.57*	α-zein precursor 19C2^CDS^
S2_160151277	8.05	darkslateblue	α-amylase/protease inhibitor^CDS^
S9_24144378	7.85	darkslateblue	Protein phosphatase 2A regulatory subunit^CDS^
S2_184267091	9.59	plum2	Flavonoid 3-monooxygenase^6.6 kb-5′^
S1_264986642	9.06	plum2	Mitogen-activated protein kinase^CDS^
S5_168853373	6.75	orange^#^	bZIP transcription factor^4.4 kb-5^
S5_168853373	9.28	138.092_70.421^#^	bZIP transcription factor^4.4 kb-5^

SNP indicates the chromosome and position (bp) within the maize genome (version 5b.60). The significance of the SNP association is indicated by the negative of the log for the false discovery rate corrected p-value. Module/marker indicates which module eigenvalue the SNP was associated. Single constituents of the midnightblue (*) and orange (^#^) modules were also analyzed by GWAS. Gene annotation describes the closest gene model relative to the SNP evaluated.

**Figure 4:**
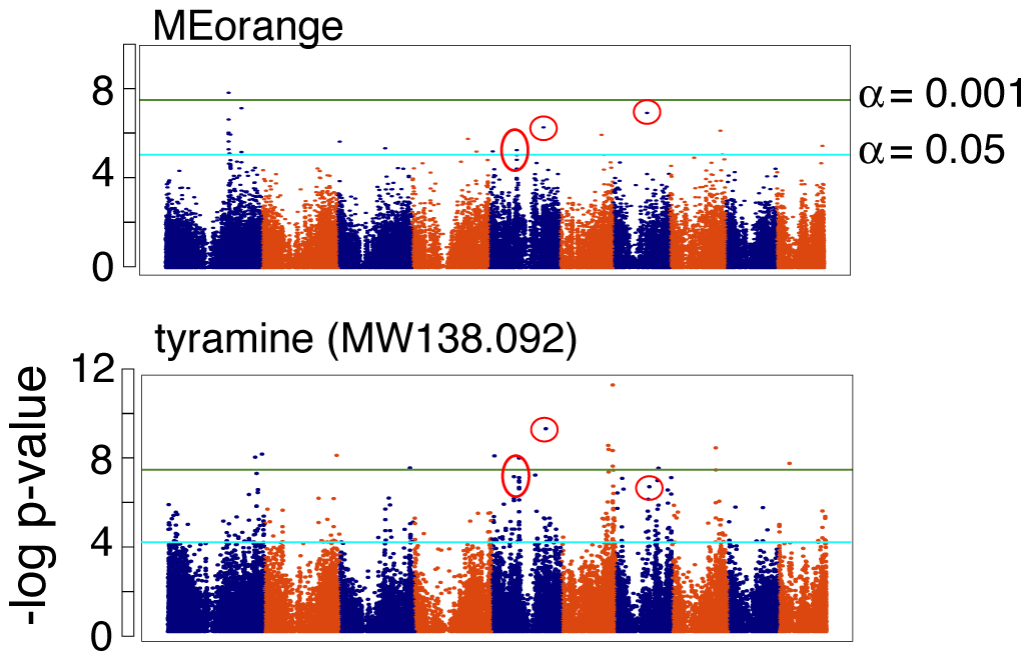
Module eigenvalues do not obscure the importance of single compounds. MEorange was estimated from 81 molecular features, one of which was identified to be tyramine. GWAS on MEorange identified 27 significant SNPs at the FDR-corrected p<0.05 threshold. GWAS on tyramine alone identified 7 SNPs in common (red circles) with MEorange.

A potential pitfall for the WGCNA procedure as a data condensation tool was the potential to excessively smooth the data, creating a false picture of the genetic regulation of the metabolome. In this instance, collapsing multiple metabolite markers into a single signal might obscure the effect of a particular locus for importance of a module constituent. To address this concern, we examined the orange module in greater detail. At 4SD, orange has 9 nodes, one of which we identified as tyramine (Figure S3). The abundance of tyramine alone was used as a trait for GWAS; this result was compared with the GWAS on the orange module eigenvalue (Figure 4). The orange module returned 27 significant SNPs, 7 of which were also identified as significant for tyramine (Table 1). While GWAS on tyramine returned more significant SNPs than for the orange module, our result does indicate that SNPs associated with a single compound can be identified from GWAS on the module eigenvalue.

### Leveraging Genomic Information to Assist Annotation of Mass Spectrometry Data

Compound identification in mass spectrometry based non-targeted metabolite profiling experiments represents a major challenge of this approach [45]. The utility of indiscriminant MS/MS (idMS/MS) was recently demonstrated to improve the rate and confidence of metabolite identification in non-targeted metabolite profiling experiments [37]. In the current study, the idMS/MS process was applied to selected features from modules with significant GWAS results. As described above, GWAS with the midnightblue module identified a region of maize chromosome 7 consistent with the α–zein 19C2 storage protein (UniProtKB P06677). Sixty-seven features were identified in this module with retention times between 226.35 and 227.06 sec, suggesting that they all represent the same compound. The reconstructed MS and idMS/MS spectra were highly suggestive of a peptide structure due the observation of multiple charge states. The molecular weight of the potential peptide was inferred from the two multiply charged isotope clusters in the MS spectra, and the corresponding idMS/MS spectrum was searched against the maize genome using the Mascot database search engine. This search returned a single peptide as the likeliest match (PAASYQQHIIGGALF), which represents the C-terminus for both the 19C1 and 19C2 variants of the α–zein storage protein. Taken together, these results suggest that *cis-*acting variation at the α-zein locus on chromosome 7 influences the quantitative expression of this protein and this variation is apparent in the cooked maize meal product. We achieved this peptide identification in spite of the fact that our MS data were collected with small molecules in mind and without the benefit of predictable proteolytic cleavage that most proteomic search engines rely upon. Further, we accomplished this from a single separation/MS experiment without the need for a second targeted MS/MS experiment, demonstrating the utility of the idMS/MS workflow. GWAS on this peptide alone returned significant SNPs common with the midnightblue module eigenvalue. However, midnightblue produced a far more significant p-value than the single feature, in contrast to the previous example (Table 1).

WGCNA alone, without the benefit of GWAS, can also assist in the annotation of mass spectra. The orange module contained 9 features with 4 different retention times at the 4SD network threshold, suggesting that there are only a few compounds contained in this module that behave similarly across this maize diversity panel. The first two eluting features demonstrated a strong similarity to tyramine, as described above. The third retention time group contained 3 isotopes of a molecular ion of 284.13, with a fragmentation patterns consistent with *p-*coumaric acid. A dehydration conjugation between tyramine and p-coumaric acid is consistent this molecular ion. The fourth retention time group contained 3 isotopes of the molecular ion 314.14; the idMS/MS patterns suggested tyramine, which lead us to hypothesize an additional tyramine conjugate. The molecular ion for 314.14 is consistent with a ferulic acid-tyramine dehydration conjugate. The final retention time group consisted of a single feature, where the idMS/MS spectra did not show strong matches in public databases. As a whole, the spectral annotations of the orange module members suggest that this cluster is focused on variation in tyramine and at least two of its phenylpropanoid conjugates. The module identification enabled annotation of the mass spectra, as we were able to restrict our decision space based on the data. Likewise, understanding the underlying chemistry should enable our analysis of the GWAS identified SNPs, to clarify how these genes would contribute to the synthesis and modification of tyramine.

### Correlation of Module Eigenvalues with Potentially Related Phenotypes

One of the original applications of WGCNA estimated eigenvalues was correlation analysis with related phenotypes, such as using patterns of gene expression to predict disease risk [46]. WGCNA was used to condense large datasets into more computationally manageable ones, with the added advantage of creating new testable hypotheses regarding cause and effect between genetic networks and phenotypic outcomes. Here, we used the WGCNA estimated module eigenvalues for stepwise regression of kernel weight, a commonly studied quality trait. GWAS was applied to kernel weight, but failed to identify any significant SNPs at the α = 0.05 significance threshold. The GWAS procedure, as implemented by GAPIT, estimated the heritability of kernel weight at 0.38, which suggested that genetic regulation of kernel weight was highly complex and resulted from the interaction of many genes of very small individual effect. Stepwise regression for kernel weight that included all module eigenvalues reduced to a model using 11 modules and explained more than half of the observed variance (Table 2). These modules included those with significant GWAS associated SNPs, modules that were well defined according to the connectivity rules, and still others that were more diffuse. While the regression model may have overestimated the fraction of variance due to genetic factors (0.548 vs. 0.38), the two analyses were consistent in their overall findings. The eigenvalue regression model summarized the contributions of 11 modules, which conservatively contained at least 600 features (Table S2). Both statistical methods indicated that kernel weight was determined by the interplay of many genetic factors, however the module regression model did quantify the relative input from defined entities and provide a logical framework from which to build additional hypotheses.

**Table 2:** Regression of kernel weight using module eigenvalues.

Source	DF	SS	F-ratio	p-value	t Ratio
MEsalmon4^gwas^	1	30242.822	22.7874	<0.0001	−4.77
MEroyalblue^gwas^	1	21356.628	16.0918	<0.0001	−4.01
MEdarkslateblue^gwas^	1	14387.453	10.8407	0.0012	3.29
MEsalmon^gwas^	1	7384.471	5.564	0.0194	2.36
MEpink^4SD^	1	36952.627	27.8431	<0.0001	5.28
MEblue^4SD^	1	20934.891	15.774	0.0001	3.97
MEdarkgrey^4SD^	1	11517.166	8.678	0.0037	2.95
MEgreen^1SD^	1	53412.158	40.245	<0.0001	−6.34
MEred^1SD^	1	31513.736	23.745	<0.0001	4.87
MEsaddlebrown^1SD^	1	20056.221	15.112	0.0001	3.89
MEwhite^1SD^	1	11504.719	8.6686	0.0037	−2.94
Error	173	229601.4			
Model	184	539795.71	21.2477	<0.0001	Adj r^2^ = 0.548

Superscripts indicate whether the module eigenvalues gave significant correlations with GWAS and/or was included in the network using the 4SD threshold or the initial description of the network (1SD threshold).

## Discussion

One of the promises of systems biology is that through the integration of analytical technologies, a more comprehensive and complete view of biological processes can be achieved. Here, we utilized mass spectrometry based non-targeted metabolite profiling to characterize the maize kernel metabolome. We chose to profile cooked maize ground in a consumer-grade grain mill to better understand the variation present in food product that might reasonably be encountered by a consumer, rather than to estimate the maximal genetic potential found in these fractions. We used WGCNA to identify the patterns that help determine composition and quality, and to resolve the multiple testing problem and rebalance the number of observations to variables under analysis. This data condensation step allowed us leverage investments made in maize genetics and genomics to assist the annotation of our mass spectra, through the application of simple (i.e. correlation and regression) and complex (GWAS) statistical procedures. While we chose to profile maize kernels, the statistical and bioinformatic process outlined here is applicable to any biological system with sufficient genetic and genomic investment and should enhance the impact of systems biology approaches in plant, animal and microbial model organisms.

A second promise of systems biology is that of translational genomics, to apply our increasingly deep view of biological processes in more applied contexts and to produce positive outcomes for society. One such application is genomic selection or whole genome prediction, where all available genetic markers are used to predict phenotypes [15]. In a recent example, a SNP microarray was used to genotype a panel of diverse maize varieties that had also been evaluated using metabolite profiling and standard agronomic evaluation [15]. Both genetic and metabolomic markers gave high accuracy predictions of agronomically important traits such as biomass accumulation and flowering time. Additionally, the use of metabolomics allowed a light to shine into the “black box” of genomic selection, where the goal is merely to apply abundant and anonymous genetic markers to predict the phenotype of interest. Through simultaneous genetic and metabolomic profiling, it is possible to correlate micro-scale phenotypes (i.e. glucose) with macro-scale phenotypes (i.e. biomass) while also generating hypotheses to address causality. The methodology we describe here is consistent with that reported by Riedelsheimer and colleagues [14], [15], although the scale of genetic and phenotypic data available to us is considerably larger.

As our understanding of biology is an accretive process, there will always be more data to include in future analyses. One of the limitations of this study is the size of the diversity panel characterized by mass spectrometry. Statistical power increases as a function of the size of the study panel, such that if we had surveyed a larger fraction of the 282 varieties we should have been able to resolve genes with smaller individual function. One of the advantages of our workflow is that it encourages reexamination (Figure 1). As we gain additional phenotypes with the study panel, we can recalculate the network and start the correlation and GWAS over again. As we identify particular metabolite or protein markers, we can apply “guilt by association” to improve the annotation of other members of the same modules and the annotation of the genome itself. SNP detection technologies are also rapidly improving in scale and price, such that repeating the GWAS in a year’s time will likely identify new regions of the maize genome that were not adequately covered in the present set of SNPs [12]. Finally, even with incomplete knowledge of the maize genome and inadequate statistical power, we were able to create a logical framework to explain an otherwise recalcitrant trait. We know that the basis of kernel weight is complicated [47], however, we can build testable hypotheses out of the module regression model that can be more fully explored in either larger diversity panels, to repeat GWAS with more power, or to choose biparental mapping populations, to test the effect of particular SNPs with the power advantages that simple mapping populations offer [48].

## Supporting Information

Figure S1 **Principal component analysis of metabolite profiling.** Clustering of technical replicates indicated good repeatability among samples. Three principal components explained 20% of the variance observed.
(TIF)

Figure S2 **Determination of** α–**zein 19C2 by MS/MS.** The reconstructed spectrum for m/z 1056.528 at 226.57 seconds revealed two strong isotope clusters at low collision energy: a cluster with a monoisotopic peak at 524.943 with isotope spacing indicative of a charge state of 3 and a second with a monoisotopic peak at 786.910 with a charge state of 2. The calculated molecular weight of the molecule was determined to by 1571.805 using the 524-isotope cluster, or 1571.804 using the 786-isotope cluster. The idMS/MS spectra were searched against the maize NCBInr protein sequence database using doubly charged 786.910 as the parent ion. The only peptide match found was that to the c-terminal peptide of α–zein 19 C1 or C2 (Mascot ion score 29, identity score 30, amino acid sequence PAASYQQHIIGGALF). This spectrum is annotated as a peptide from the α-zein 19 C protein with identification confidence level 2.
(TIF)

Figure S3 **Determination of tyramine by MS/MS.** The reconstructed idMS/MS spectrum for m/z 138.092 at 70.4 seconds demonstrates strong similarity to the NIST MS/MS spectrum for Tyramine, matching fragments 138.09 and 121.058 with high mass accuracy. This feature is annotated as tyramine with identification confidence level 2.
(TIF)

Table S1 **Data matrix of maize genotypes and metabolomic features.** This table describes the subset of the Buckler Maize Diversity Panel profiled using non-targeted UPLC-MS/MS and the 8,710 features detected by the XCMS software. (21.9 Mb.csv file).
(ZIP)

Table S2 **Description of modules at three connectivity (significance) levels and results of genome wide association studies on module eigenvalues.** At the ≥1SD connectivity threshold, 97.5% of features detected by UPLC-MS/MS are included in the network. At ≥4SD, 47.1% of features detected are included. At ≥6SD, 20.8% of features detected are included. Using the module eigenvalues estimated from all 56 modules (≥1SD threshold), GWAS returned significantly associated SNPs for 19 modules (33.9%).
(DOCX)

File S1 **MS and MS/MS spectra for tyramine, coumaryl tyramine, feruloyl tyramine, and 19C2 alpha zein.**
(MSPLIB)
