# Supplementary figures and images for: Leveraging Non-Targeted Metabolite Profiling via Statistical Genomics
Source: PLoS One. 2013 Feb 28;8(2):e57667. doi: 10.1371/journal.pone.0057667 (PMC3585405; doi:10.1371/journal.pone.0057667)

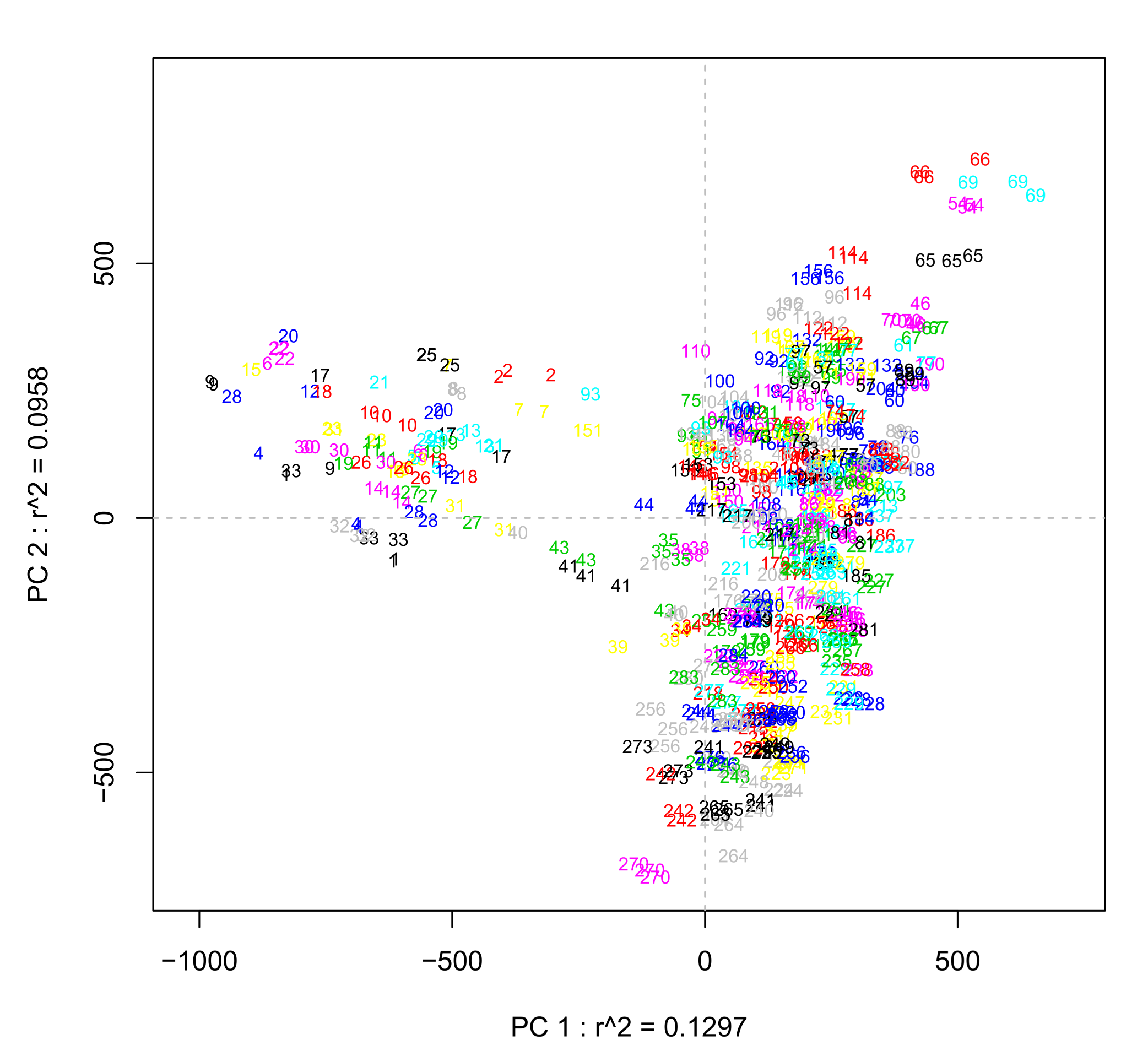

Supplement: Figure S1 — Principal component analysis of metabolite profiling. Clustering of technical replicates indicated good repeatability among samples. Three principal components explained 20% of the variance observed. (TIF) [file pone.0057667.s001.tif]

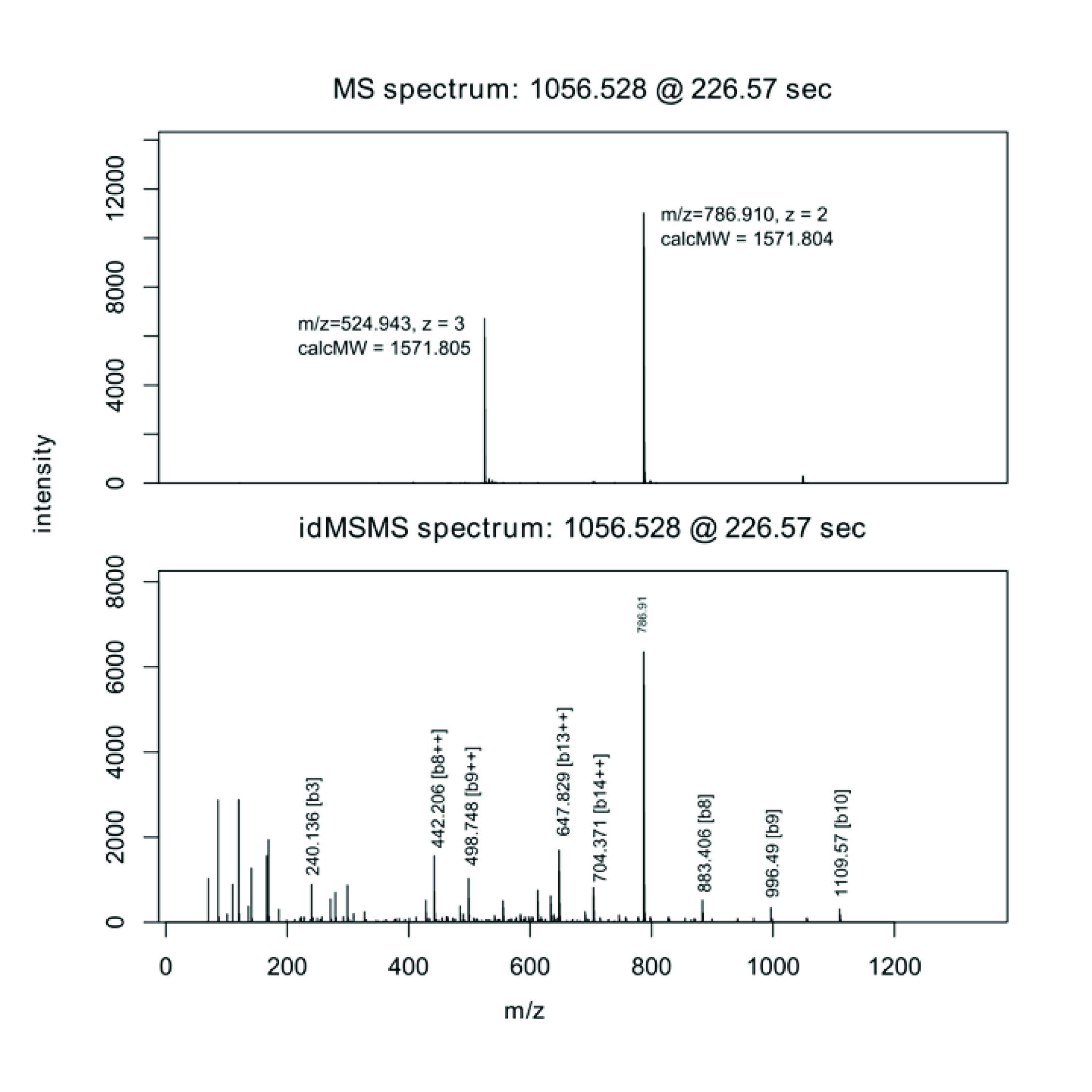

Supplement: Figure S2 — Determination of α–zein 19C2 by MS/MS. The reconstructed spectrum for m/z 1056.528 at 226.57 seconds revealed two strong isotope clusters at low collision energy: a cluster with a monoisotopic peak at 524.943 with isotope spacing indicative of a charge state of 3 and a second with a monoisotopic peak at 786.910 with a charge state of 2. The calculated molecular weight of the molecule was determined to by 1571.805 using the 524-isotope cluster, or 1571.804 using the 786-isotope cluster. The idMS/MS spectra were searched against the maize NCBInr protein sequence database using doubly charged 786.910 as the parent ion. The only peptide match found was that to the c-terminal peptide of α–zein 19 C1 or C2 (Mascot ion score 29, identity score 30, amino acid sequence PAASYQQHIIGGALF). This spectrum is annotated as a peptide from the α-zein 19 C protein with identification confidence level 2. (TIF) [file pone.0057667.s002.tif]

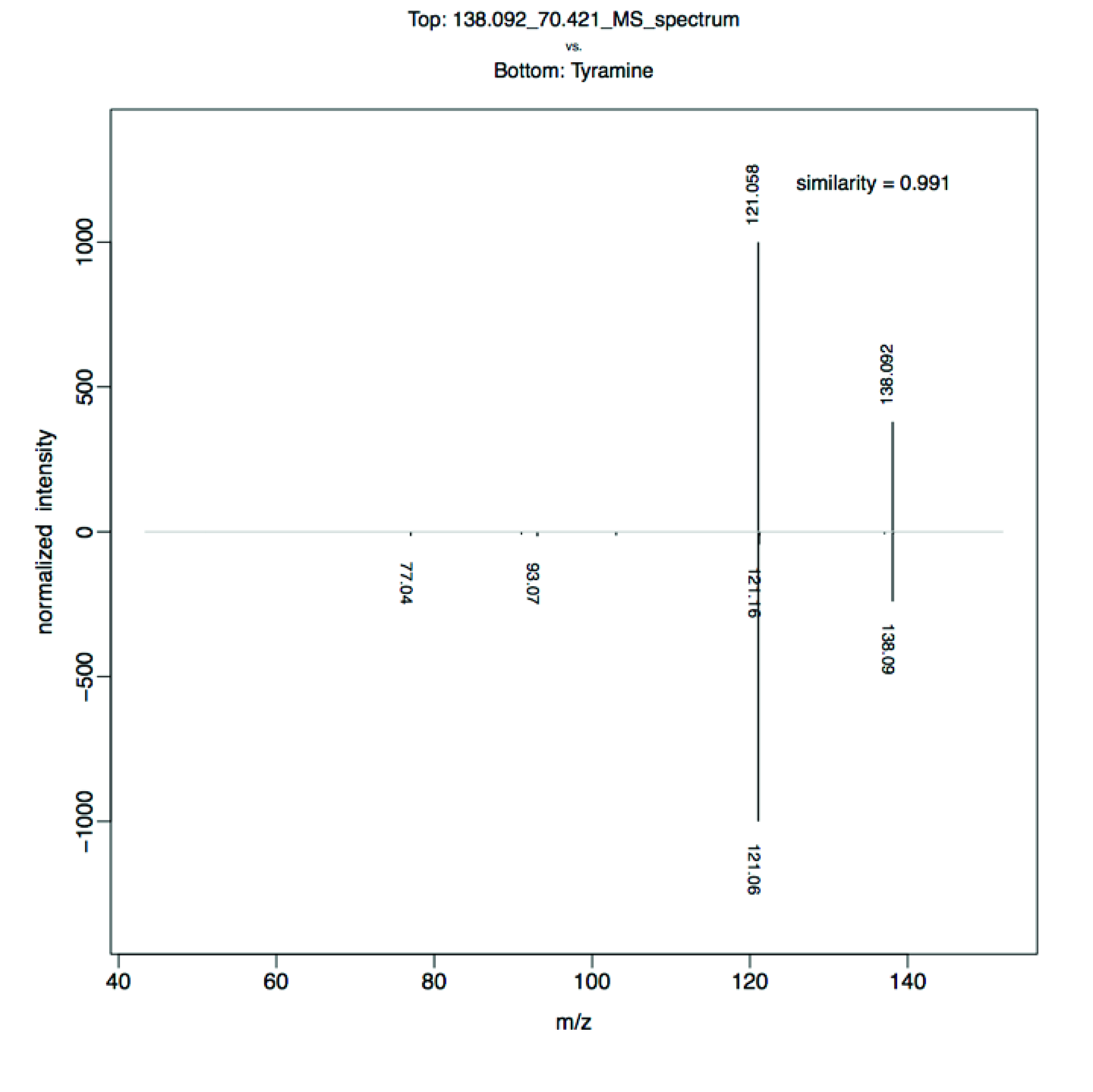

Supplement: Figure S3 — Determination of tyramine by MS/MS. The reconstructed idMS/MS spectrum for m/z 138.092 at 70.4 seconds demonstrates strong similarity to the NIST MS/MS spectrum for Tyramine, matching fragments 138.09 and 121.058 with high mass accuracy. This feature is annotated as tyramine with identification confidence level 2. (TIF) [file pone.0057667.s003.tif]
