# Supplementary material for: Leveraging Non-Targeted Metabolite Profiling via Statistical Genomics
Source: PLoS One. 2013 Feb 28;8(2):e57667. doi: 10.1371/journal.pone.0057667 (PMC3585405; doi:10.1371/journal.pone.0057667)
Supplement: Table S2 — Description of modules at three connectivity (significance) levels and results of genome wide association studies on module eigenvalues. At the ≥1SD connectivity threshold, 97.5% of features detected by UPLC-MS/MS are included in the network. At ≥4SD, 47.1% of features detected are included. At ≥6SD, 20.8% of features detected are included. Using the module eigenvalues estimated from all 56 modules (≥1SD threshold), GWAS returned significantly associated SNPs for 19 modules (33.9%). (DOCX) [file pone.0057667.s005.docx]

| **Module Name** | **Network @ ≥ 1SD**  **Nodes;Edges (Modules)** | **Network @ ≥ 4SD**  **Nodes;Edges** | **Network@ ≥6 SD**  **Nodes;Edges** | **GWAS SNP associations** |
| --- | --- | --- | --- | --- |
| Bisque4 | 40;13284 | 6;24 |  |  |
| Black | 260;231845 | 49;2082 |  |  |
| Blue | 653;701931 | 67;1978 |  |  |
| Brown | 1009;885309 | 622;354403 | 567;199538 |  |
| Brown4 | 109;78871 | 98;12732 | 8;199 |  |
| Cyan | 142;22313 | 21;93 | 5;18 |  |
| Darkgreen | 78;24236 |  |  |  |
| Darkgrey | 340;482129 | 275;49604 | 21;50 |  |
| Darkmagenta | 54;2241 | 3;4 |  |  |
| Darkolivegreen | 62;43177 | 23;1713 |  |  |
| Darkorange | 69;14123 | 30;597 | 14;98 |  |
| Darkorange2 | 41;2413 | 9;34 |  | 5 |
| Darkred | 83;10075 | 42;246 |  | 230 |
| Darkslateblue | 36;4199 | 20;169 | 5;18 | 401 |
| Darkturquoise | 66;6825 | 6;16 |  |  |
| Floralwhite | 40;2024 |  |  |  |
| Green | 324;74401 |  |  |  |
| Greenyellow | 156;7261 | 19;70 | 5;18 |  |
| Grey | 44;234 | 31;82 | 20;54 | 56 |
| Grey60 | 112;18663 |  |  |  |
| Honeydew | 29;4103 | 12;32 |  |  |
| Ivory | 40;5337 |  |  |  |
| Lavenderblush | 28;594 | 18;214 | 4;6 | 6 |
| Lightcyan | 138;35700 | 63;2066 | 17;110 |  |
| Lightcyan1 | 42;8358 | 21;154 |  |  |
| Lightgreen | 98;32270 | 46;1148 |  |  |
| Lightpink | 32;6405 | 4;8 |  |  |
| Lightsteelblue | 41;2909 | 21;392 | 17;158 | 46 |
| Lightyellow | 87;21029 | 4;8 |  |  |
| Magenta | 231;53595 | 81;2058 | 7;12 |  |
| Maroon | 30;6214 | 2;2 |  | 49 |
| Mediumpurple | 46;1413 | 22;144 | 4;12 | 59 |
| Midnightblue | 138;28187 | 108;4622 | 55;1324 | 25 |
| Navajowhite | 35;15199 |  |  |  |
| Orange | 63;7098 | 9;56 |  | 27 |
| Orangered | 48;42969 | 2;42 |  |  |
| Paleturquoise | 63;4986 | 20;164 |  |  |
| Paleviolet | 36;43565 | 33;4880 | 8;15 |  |
| Pink | 259;62809 | 61;936 |  |  |
| Plum | 50;8817 | 17;147 |  |  |
| Plum2 | 36;1050 | 28;298 | 22;142 | 2169 |
| Purple | 189;151892 | 185;83179 | 174;41641 |  |
| Royalblue | 82;8058 | 32;356 |  | 48 |
| Saddlebrown | 64;11486 |  |  |  |
| Salmon | 151;30863 | 104;7882 | 79;3842 | 1128 |
| Salmon4 | 35;7657 | 25;422 | 18;72 | 11 |
| Sienna3 | 58;5772 | 34;792 | 27;392 | 178 |
| Skyblue | 64;4053 | 20;176 | 13;36 | 309 |
| Skyblue3 | 55;3668 | 32;532 | 21;336 |  |
| Steelblue | 60;3301 | 48;694 | 22;110 | 41 |
| Tan | 160;34955 | 51;1676 | 31;500 |  |
| Thistle1 | 35;15643 |  |  |  |
| Thistle2 | 31;436 | 18;122 | 4;6 | 24 |
| Turquoise | 2105;3738652 | 1597;626531 | 635;40217 |  |
| Violet | 58;26353 | 43;1175 | 10;18 | 19 |
| Yellowgreen | 54;5056 | 20;116 |  |  |
| **Totals** | 8489;7066006 (56) | 4102;1164870 (48) | 1813;288942 (27) | 4830 (19) |

**Table S2: Description of modules at three connectivity (significance) levels and results of genome wide association studies on module eigenvalues.**  At the ≥ 1SD connectivity threshold, 97.5% of features detected by UPLC-MS/MS are included in the network. At ≥ 4SD, 47.1% of features detected are included. At ≥ 6SD, 20.8% of features detected are included. Using the module eigenvalues estimated from all 56 modules (≥ 1SD threshold), GWAS returned significantly associated SNPs for 19 modules (33.9%).
